# Acute non‐heparin‐induced thrombocytopenia during hemodiafiltration in a patient with multiple myeloma

**DOI:** 10.1002/ccr3.1997

**Published:** 2019-02-22

**Authors:** Makiko Morita, Kayoko Nakanishi, Kenta Masuda, Kazuhiro Yoshida, Daiki Shimomura, Atsumi Ishida, Shuichi Shiga, Satoshi Ichiyama

**Affiliations:** ^1^ Department of Clinical Laboratory Kyoto University Hospital Kyoto Japan; ^2^ Artificial Kidney Unit Kyoto University Hospital Kyoto Japan; ^3^ Department of Laboratory Medicine Tenri Hospital Nara Japan

**Keywords:** bortezomib, hemodiafiltration, hemodialysis, heparin‐induced thrombocytopenia, platelet aggregation

## Abstract

This report demonstrates that not only heparin‐induced thrombocytopenia, but also hemodialysis conditions (platelet activation due to hemodiafiltration and heparin underdosing) may markedly reduce the platelet count and cause clotting in the hemodialysis circuit in patients in a hypercoagulable state. The clot prevention effects of bortezomib are therefore of great importance.

## 1 INTRODUCTION

Heparin‐induced thrombocytopenia (HIT) is a potentially fatal adverse effect caused by heparin and is characterized by declining platelet counts after 5‐10 days of heparin therapy.^1^, ^2^ HIT is caused by antibodies generated against heparin‐platelet factor 4 (PF4) complexes that result in platelet activation and aggregation.^3^ Laboratory immunological assays detecting HIT antibodies (ie, enzyme‐linked immunosorbent assays and latex immunoturbidimetric assays) have excellent sensitivity, and a negative result can exclude HIT from differential diagnosis.^4^, ^5^, ^6^ Therefore, it is essential to investigate other causes for thrombocytopenia when the results for the HIT antibody are negative.

Here, we report a case in which a 71‐year‐old woman with multiple myeloma presented with repeated hemodialysis (HD) circuit clotting and sudden thrombocytopenia after hemodiafiltration (HDF) with heparin (unfractionated heparin; UFH) (platelet count from 234 × 10^9^/L in pre‐HDF to 27 × 10^9^/L in post‐HDF) despite obtaining negative results from a HIT antibody test.

## 2 CASE REPORT

**Figure 1:**
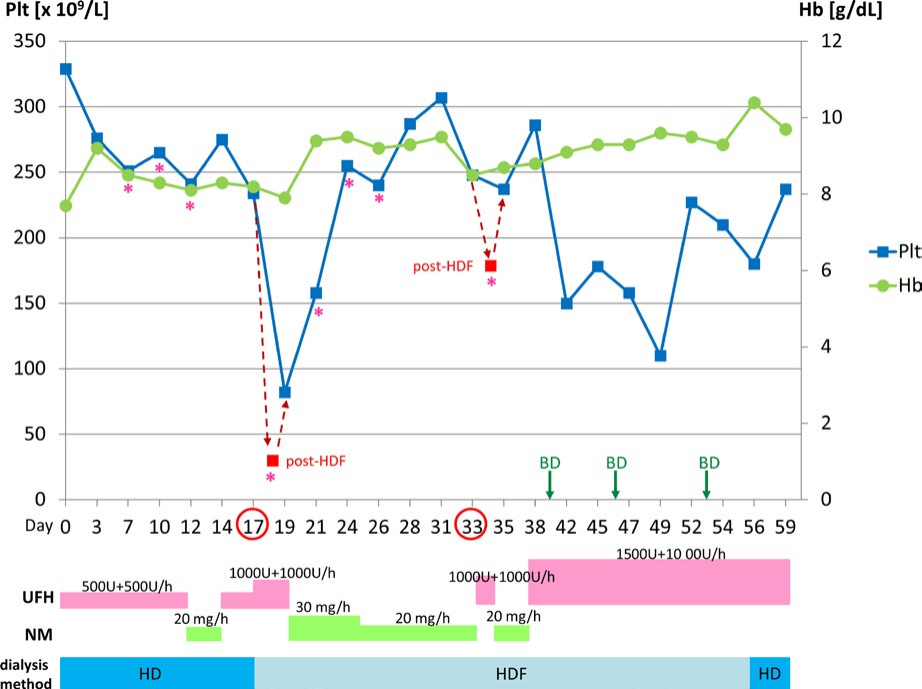
Time course of platelet counts (blue) and hemoglobin concentration (green) during hemodialysis (HD) or hemodiafiltration (HDF) with anticoagulants (UFH, unfractionated heparin; NM, nafamostat mesilate) and their dosage. Blood samples for hematological examinations were obtained before HD/HDF except for day 17 and 33 (obtained pre‐ and post‐HDF, indicated by the red square). Samples for anti‐PF4/heparin antibodies were obtained post‐HDF on day 17. Asterisk (*) indicates clotting in the dialysis circuit. Red circles on the horizontal axis indicate the day that platelet decline was observed after HDF. BD indicates bortezomib‐dexamethasone treatment

A 71‐year‐old woman suspected of a right iliac metastatic tumor was referred to our hospital. Laboratory examinations suggested multiple myeloma with the following results: Hb, 7.7 g/dL; CRE, 6.60 mg/dL; BUN, 76 mg/dL; eGFR, 5.4 mL/min/1.73m^2^; Ca, 9.2 mg/dL; FLC κ, 9660 mg/L; FLC λ, 18.40 mg/L; FLC κ/λ ratio, 525; urine Bence Jones Protein (BJP‐κ), positive. Normal immunoglobulins were suppressed by drastic increases of free light chain κ with the following results: IgG, 576 mg/dL; IgA, 36 mg/dL; IgM, 16 mg/dL. Other results were as follows: WBC, 6.73 × 10^9^/L; Plt, 329 × 10^9^/L; PT%, 95%; aPTT, 30.6 seconds; Fib, 478 mg/dL; d‐dimer, 7.1 µg/mL. No medications were taken at the time of admission. A bone marrow aspiration test revealed the presence of monoclonal plasma cells (CD38+ Cytoplasmic‐κ+, DNA aneuploidy [56 chromosomes]). No megakaryocytic dysplasia or megakaryocytopenia was observed in the marrow. For the treatment of renal impairment, HD with heparin as an anticoagulant was initiated on the admission day with a bolus of 500 U at the start of the session followed by a maintenance infusion of 500 U/h. The time course of the platelet count and detailed information regarding the HD are shown in Figure 1. On day 12, the anticoagulant was temporarily changed to nafamostat mesilate (NM) to prevent bleeding during a bone marrow aspiration test scheduled on the same day. Anticoagulation using heparin at the same dose was restarted on day 14, and on day 17, the bolus dose was increased to 1000 U and 1000 U/h for maintenance since clotting in the HD circuit was observed during previous HD sessions. The dialysis method was also changed to postdilutional HDF (TDF‐15M; Toray Medical, Co., Ltd., Tokyo, Japan) for the purpose of free light chain removal. Clotting in the circuit was observed even after increasing the heparin dose, and post‐HDF laboratory examinations revealed a marked reduction in platelet count from 234 × 10^9^/L to 27 × 10^9^/L. The aPTT was normal (32.3 seconds). No red cell fragments were observed on the peripheral blood smear. We did not observe the sudden onset of anemia based on the hemoglobin levels shown in Figure 1. Since we suspected HIT, anticoagulation with NM was subsequently initiated. The 4Ts score proposed by Warkentin^7^, ^8^, ^9^ had a total of 4 points (intermediate): 2 points for thrombocytopenia, 1 for the timing of platelet count fall, 0 for thrombosis, and 1 for other causes of thrombocytopenia^10^ (anemia, primary hematologic disorder, and elevated d‐dimer score). The discontinuation and initiation of heparin and NM, respectively, resulted in plate count normalization. Although clotting was observed during HDF with NM, it was resolved by changing the dialysis catheter. On day 33, during HDF with NM, the results from a HIT antibody test by latex immunoturbidimetric assay using HemosIL HIT‐Ab (PF4‐H) (Instrumental Laboratory, Japan) were negative. Therefore, anticoagulation using heparin was restarted using a bolus dose of 1000 and 1000 U for maintenance. However, since clotting in the hemofilter reoccurred, anticoagulation with NM was reinitiated. The platelet count also dropped from 248 × 10^9^/L to 186 × 10^9^/L after HDF. She eventually received HDF with high‐dose heparin at 1500 U for bolus and 1000 U/h for maintenance from day 38. Chemotherapy with bortezomib and dexamethasone (BD) was initiated on day 39 and was administered once a week thereafter (day 39, 46 and 53). Of note, no unexpected clotting events occurred during BD treatment and high‐dose heparin anticoagulation. Since her condition improved (FLC‐κ 23.2 mg/L on day 59), she was transferred to another hospital on day 59 for HD maintenance and further treatment.

## 3 DISCUSSION

Heparin‐induced thrombocytopenia is a major cause of sudden thrombocytopenia for patients undergoing HD and occurs in up to 17.4% of patients receiving HD with heparin.^11^ We experienced repeated HD circuit clotting and non‐HIT acute thrombocytopenia after HDF and investigated its possible mechanism. We speculate that the marked drop in platelet count and clotting in the HD circuit were attributed to the hypercoagulable state of the patient that was induced by myeloma, heparin underdosing, and other HD conditions.

Patients with MM are in a hypercoagulable state since factor VIII (FVIII) activity and von Willebrand factor (vWF) antigen are highly elevated and protein S levels are decreased.^12^, ^13^, ^14^ Furthermore, these patients have an increased risk of venous thromboembolism (VTE).^15^, ^16^ FVIII activity is correlated with a higher incidence of VTE.^17^ The production of procoagulant auto‐antibodies with prothrombotic properties has also been reported in MM patients.^18^

During HD or HDF, the exposure of blood to the hemofilter surface is inevitable. Therefore, it is important to minimize the biological reaction by choosing the appropriate hemodialysis condition, especially for patients with hypercoagulable states. Platelet‐derived microparticles have been reported to significantly increase after HDF^19^ and accelerate thrombin generation.^1^ Moreover, Sirolli et al^20^ reported that polysulfone membrane (as used in our case) can be prothrombotic since it increases circulating activated platelets and platelet‐erythrocyte aggregation. Additionally, postdilutional HDF may accelerate coagulation activation since it has potent dehydration abilities. Although a blood sample for HIT antibody examination was taken after HDF in this case, trapping of the antibody by the HDF membrane was ruled out because the molecular weight of IgG was larger than the filtering capacity of the membrane.

Nafamostat mesilate, a serine protease inhibitor, has potent inhibitory effects on thrombin, coagulation factor XIIa, factor Xa, factor VIIa, kallikrein, plasmin, compliment factors, and trypsin.^21^ Furthermore, NM inhibits platelet aggregation and disaggregates already aggregated platelets.^21^ Fuse et al^22^ have suggested that NM suppresses activated glycoprotein IIb‐IIIa expression. The reason that platelet reduction was not observed during NM use may be attributed to its potent inhibitory effect on coagulation and platelet aggregation. The clotting that occurred during HDF with NM observed on day 21, 24, and 26 may have been caused by catheter malposition or catheter occlusion since it was resolved by changing the catheter.

Bortezomib and dexamethasone is frequently used for the treatment of MM. Bortezomib and dexamethasone has inhibitory effects on platelet aggregation induced by ADP, ristocetin, and collagen.^23^, ^24^, ^25^ Dexamethasone accelerates platelet aggregation by suppressing cyclooxygenase (COX)‐2 expression^26^, ^27^ that regulates prostacyclin (prostaglandin I2; PGI2) production^28^ which attenuates platelet aggregation.^29^ Since bortezomib reduces the risk for VTE,^30^ its antithrombotic effect is more potent than the prothrombotic effect of dexamethasone. These inhibitory effects on coagulation and platelet activation by bortezomib correspond with the observation that clotting in the dialysis circuit did not occur during BD treatment from day 39‐59. In patients with MM, nuclear factor‐kappa B (NF‐κB) is constitutively activated and leads to the antiapoptosis and proproliferation of myeloma cells.^31^ BD have antitumor effects by suppressing NF‐κB,^32^, ^33^ a protein complex that is associated with platelet budding from megakaryocytes,^34^ thereby inducing the reduction in platelet production. This was consistent with the course seen from day 39.

As prolongation of aPTT was not observed after HDF on day 17, we excluded the possibility of sudden onset acquired VWD mimicking VWD type 2B due to platelet activation by the dialysis membrane.

In summary, we speculate that the repeated hemofilter clotting and platelet count reduction were caused by three possible factors: a hypercoagulable state induced by MM, platelet activation by HDF, and heparin underdosing. Therefore, we emphasize the importance of careful observation of the dialysis condition and hematological examinations, especially for cancer patients with hypercoagulable states.

## CONFLICTS OF INTEREST

The authors declare no conflicts of interest.

## AUTHOR CONTRIBUTION

MM: examined the patient's samples, analyzed the data and wrote the paper. KN, KM, DS, and AI: analyzed the data and edited the manuscript. KY: advised on dialysis. SS and SI: made intellectual contributions. All authors have approved the final manuscript.
